# Effects of Systemic Pesticides Imidacloprid and Metalaxyl on the Phyllosphere of Pepper Plants

**DOI:** 10.1155/2013/969750

**Published:** 2013-06-09

**Authors:** Constantinos Moulas, Christos Petsoulas, Konstantina Rousidou, Chiara Perruchon, Panagiotis Karas, Dimitrios G. Karpouzas

**Affiliations:** University of Thessaly, Department of Biochemistry and Biotechnology, Ploutonos 26 and Aiolou Street, 41221 Larisa, Greece

## Abstract

Microbes inhabiting the phyllosphere of crops are exposed to pesticides applied either directly onto plant foliage or indirectly through soil. Although, phyllosphere microbiology has been rapidly evolving, little is still known regarding the impact of pesticides on the epiphytic microbial community and especially on fungi. We determined the impact of two systemic pesticides (metalaxyl and imidacloprid), applied either on foliage or through soil, on the epiphytic fungal and bacterial communities via DGGE and cloning. Both pesticides induced mild effects on the fungal and the bacterial communities. The only exception was the foliage application of imidacloprid which showed a more prominent effect on the fungal community. Cloning showed that the fungal community was dominated by putative plant pathogenic ascomycetes (Erysiphaceae and *Cladosporium*), while a few basidiomycetes were also present. The former ribotypes were not affected by pesticides application, while selected yeasts (*Cryptococcus*) were stimulated by the application of imidacloprid suggesting a potential role in its degradation. A less diverse bacterial community was identified in pepper plants. Metalaxyl stimulated an Enterobacteriaceae clone which is an indication of the involvement of members of this family in fungicide degradation. Further studies will focus on the isolation of epiphytic microbes which appear to be stimulated by pesticides application.

## 1. Introduction

Phyllosphere is the habitat of a diverse microbial community dominated by bacteria, fungi, and yeasts while archaea are not particularly abundant [1]. Until recently most studies on the microbiology of the phyllosphere had focused on the ecology and interactions of microbial plant pathogens with the plant, whereas little was known regarding the role and ecology of nonplant pathogenic microorganisms on plant phyllosphere. It is now well documented that epiphytic microorganisms could serve significant functional roles including (a) suppression of plant pathogens in the phyllosphere of agricultural crops [2], (b) nitrogen fixation [3], (c) methanol utilization [4], and (d) degradation of organic pollutants [5]. In addition, microbial interactions on plant phyllosphere have been found to determine colonization of edible parts of plants by human pathogens [6]. This is particularly important for the consumption of fresh salad, fruits, and vegetables.

In a pioneering study, Yang et al. [7] demonstrated that the microbial diversity on plant phyllosphere is much higher than what had been estimated before based on culture-dependent methods but it is still lower than the microbial diversity in rhizosphere or even bulk soil [8]. Phyllosphere is an oligotrophic environment with patchy distribution of C sources where microorganisms are exposed to stress conditions including extreme exposure to UV radiation, violent fluctuations of temperature, and limited water availability [9]. In order to survive under these conditions, phyllosphere microorganisms have developed various mechanisms including pigmentation [10], DNA repair mechanisms [11], production of biosurfactants [12], and extracellular polymeric substances [13].

Apart from the abiotic and biotic stress conditions described, microorganisms on the phyllosphere of cultivated plants are exposed to pesticides. There is a wealth of literature regarding the impact of pesticides on soil microorganisms [14]. This is not surprising considering that soil constitutes largely the final deposit of both foliar and soil-applied pesticides. However, only a few studies so far have addressed the impact of pesticides onto nontarget microorganisms on plant phyllosphere. A series of studies by Zhang et al. [15–17] showed that the insecticides cypermethrin and abamectin induced changes in the structure of the bacterial community in pepper, cucumber, and broccoli phyllosphere. In a similar study the application of the fungicide enostroburin induced substantial changes in bacterial community in wheat phyllosphere [18]. All those studies have focused on potential effects on the bacterial community after foliar application of pesticides. However, only limited data are available regarding pesticides effects on nontarget fungi inhabiting plant phyllosphere. Apart from foliar application, systemic pesticides are commonly applied via soil drenching and they are translocated through the phloem to the aerial parts of the plants offering protection from pest and pathogens. However, nothing is known regarding the impact of such soil applications on the epiphytic microbial community.

Imidacloprid is a systemic insecticide which has gained registration for 140 uses in 120 countries [19]. It is applied either directly on the foliage or via soil drenching for the control of aphids (*Myzus persicae, Myzus nicotianae*), white fly (*Trialeurodes vaporariorum*), and Colorado beetle (*Leptinotarsa decemlineata*) in fruits crops, vegetables, and potatoes. Metalaxyl-M is a systemic fungicide which is applied either on foliage or via soil drenching for the control of oomycetes such as *Phytophthora parasitica* (tobacco), *Phytopthora infestans* (potato), and *Pythium* sp. [20]. Nothing is known regarding the impact of those pesticides on the microbial community of plant phyllosphere.

We aimed to investigate the impact of the systemic pesticides imidacloprid and metalaxyl on the fungal and bacterial communities on the phyllosphere of pepper plants. The influence of the mode of application, foliar versus soil application, on the magnitude and type of effects was also determined via denaturing gradient gel electrophoresis (DGGE) and cloning.

## 2. Materials and Methods

### 2.1. Experimental Setup

Three-week-old pepper plants (*Capsicum annum* L. cv Ozho) (kindly supplied by AgriPlant A.S.) were initially transplanted into 3 L plastic pots which had been filled with appropriate amounts of a 1 : 1 mixture of sand and soil (sandy, pH 7.81, electrical conductivity 0.017 mmhos cm^−1^, organic matter content 8.8 g kg^−1^, P-Olsen 2 mg g^−1^, K 215 mg g^−1^, and Mg 265 mg g^−1^). In total 18 pots were prepared and placed randomly in the growth chamber at 22°C using a 16 h light/8 h night period. The plants were watered as needed and 30 mL of Hoagland solution [21] was applied twice weekly. Pepper plants were grown under these conditions for a week and then transferred to a commercial greenhouse situated in the area of Velestino, Magnesia, Greece, where the experiment was contacted. The plants were left in the greenhouse for a period of three weeks to become acclimatized and allow the development of a natural phyllosphere microbial community as much as possible. During the acclimation period the pepper plants were watered and fertilized as needed. 

At the end of the acclimation period the 18 pots with the pepper plants were divided into 6 groups of three. The first three pots received a foliage treatment with an aqueous suspension of the insecticide imidachloprid (CONFIDOR, 200SL), while the next three pots received a soil drenching with an aqueous suspension of the same insecticide. Similarly, the next two groups of pots received a foliar or a soil drenching application of the fungicide metalaxyl (RIDOMIL GOLD, 46.52SL). The application rates in both foliar and soil applications were as suggested for the control of the target pests and diseases. Finally, the remaining two groups of pots received the same volume of water applied through foliar or soil application of the two pesticides to serve as untreated controls. Five days after pesticide applications 10 leaves per plant were collected and placed into sterile plastic bags and transported on ice to the laboratory where they were stored at −20°C until further used.

### 2.2. DNA Extraction

The microbial DNA of the phyllosphere was extracted as described by Yang et al. [7] with slight modifications. Briefly, leaf samples were transferred aseptically into polypropylene tubes containing 0.1 M potassium phosphate buffer (pH 7.0) and sonicated for 10 min in an ultrasonic bath to dislodge microorganisms from the leaf surface. The leaf remains were removed by a mild centrifugation step (3 min 500 × g) and the clear suspension was subjected to centrifugation at 7000 × g for 15 min. The supernatant was removed and the microbial pellet obtained was used for DNA extraction using the NucleoSpin Tissue kit (Macherey-Nagel, Germany) according to manufacturers' extraction.

### 2.3. PCR-DGGE Analysis

For studying the bacterial community, a nested PCR amplification of the 16S rRNA gene was used. In the first PCR round, DNA was amplified with universal bacterial primers 63f-1087r (*ca*. 1000 bp). The product obtained (1 mL) was nested with primers 357f+GC and 534r which amplify a 194 bp fragment of the 16S rRNA gene, including the variable V3 region. A 40 bp GC clamp at the 5 end of primer 357f was used [22]. For studying the fungal community, DNA was amplified with primers ITS1F-ITS4 (*ca*. 600 bp) [23]. The products obtained were used as templates (1 mL) for a second semi nested PCR with the primers ITS1F + GC and ITS2 (*ca*. 300 bp). Thermocycling conditions and the concentrations of the reagents used were as described elsewhere [24]. 

DGGE analyses were carried out on an INGENYphorU-2x2 system (Ingeny International BV, The Netherlands). Polyacrylamide gels (8%) in 1 × TAE buffer (40 mM Tris base, 20 mM acetic acid, and 1 mM disodium EDTA, pH 8.2) were prepared. The polyacrylamide gels were made with denaturating gradient of 30–55% and 50–60% for DGGE profiling of the fungal and bacterial communities, respectively (where 100% denaturant contains 7 M urea and 40% formamide). The electrophoresis was run for 16 h at 60°C and 75 V and gels were silver stained. The image was captured using a digital camera and subsequent analysis was performed with Cross Checker 2.9 v (Wageningen University, The Netherlands). Binary data for the presence/absence of bands in all samples were derived and used for statistical analysis.

### 2.4. Clone Libraries

Clone libraries for both communities were constructed based on the fragments generated by the first PCR step. Since the results showed that replicate samples of the same treatment showed minimum variability, the triplicate PCR products from the same treatment were pooled and purified/concentrated to a final volume of 30 *μ*L using the NucleoSpin II PCR clean-up kit (Macherey-Nagel GmbH, Germany). Cloning into the pGEM-T vector (Promega, Madison, USA) was performed as described by Sambrook et al. [25]. Subsequent screening of the clone libraries by PCR and DGGE was carried out as described by Liang et al. [26]. Thirty-five white colonies were selected for each treatment and were subjected to colony PCR using primers 357f+GC-534r and ITS1F+GC-ITS2 for bacterial and fungal libraries, respectively. Positive clones were screened on a DGGE gel to determine their electrophoretic mobility compared with the band pattern of the original environmental sample. Representative clones for each band type matching the migration pattern of bands in the original samples were sequenced. In cases where several clones showed identical migration pattern with a single DGGE band, three clones or more were sequenced in order to check for possible comigration of diverse sequences. For sequencing, plasmid DNA was extracted and purified from selected colonies using the NucleoSpin Plasmid kit (Macherey-Nagel GmbH, Germany) and sent for sequencing. Sequences were deposited in the European Molecular Biology Laboratory (EMBL) database and their accession numbers are HF947094-HF947095 and HF947030-HF947093 for bacterial and fungal clones, respectively.

### 2.5. Statistical Analysis

The binary data matrices obtained for each DGGE gel were used for multivariate statistical analysis to compare the effect of pesticides and their mode of application on the structure of the microbial communities on phyllosphere. Dendrograms from Jaccard distance matrices using the group average algorithm were prepared using the MultiVariate Statistical Package (MVSP) 3.13v software (http://www.kovcomp.com/).

## 3. Results

### 3.1. Effects of Pesticides on the Fungal Community

**Figure 1:**
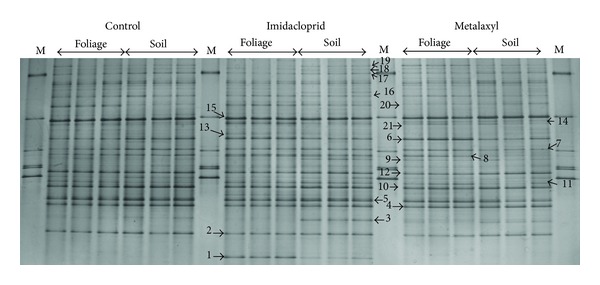
DGGE analysis of the fungal community in the phyllosphere of pepper plants subjected to foliage or soil applications of imidachloprid, metalaxyl, or water (untreated control). Lanes designated with M correspond to a marker which contained 20 ng *μ*L^−1^ of the ITS-PCR products of the following fungi with the sequence they appear on the gel from top to bottom: *Pleurotus djamor, Fusarium oxysporum* f.sp. *radicis-lycopersici*, *F. solani, P. eryngii, P. ostreatus*, and* P. cystidiosus.* Bands identified through screening with clone libraries are designated with arrows accompanied with a code number as shown in Table 1.

**Figure 2:**
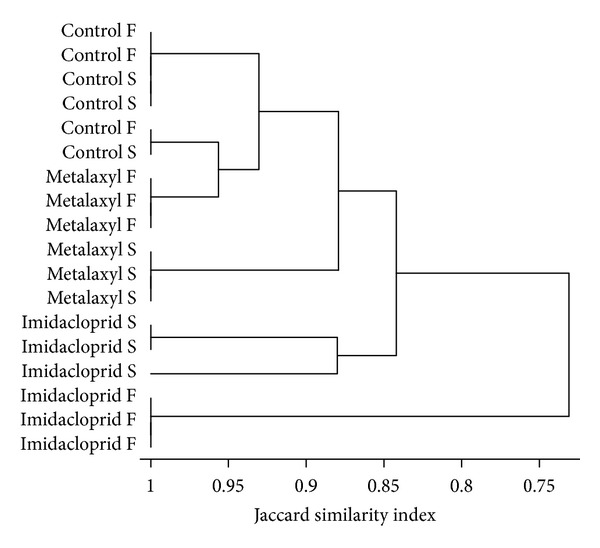
Cluster analysis (group average, Jaccard similarity index) of the banding patterns generated by DGGE fingerprinting analysis of the fungal community.

The fingerprints produced by the three replicates of the same treatment were highly similar (Figure 1). Overall, DGGE analysis of the fungal community provided rather complex banding patterns with band numbers exceeding 20 in all treatments. Cluster analysis of the DGGE banding patterns resulted in the development of two main clusters (Figure 2). The first main cluster comprised all control and metalaxyl-treated samples along with the samples which received a soil application of imidacloprid. Samples contained within the first cluster shared a similarity of >84%. The second cluster was composed only by the samples which received a soil application of imidacloprid and showed >70% similarity with the samples of the first cluster. In the first cluster, samples were further separated according to the pesticide applied with soil-treated samples of imidacloprid separated from the metalaxyl-treated samples and the controls which grouped together. Within the latter subcluster the foliage-treated metalaxyl samples and the control samples clustered together (>90% similarity) while the soil-treated metalaxyl samples were separated.

Clone libraries were developed to identify the main members of the fungal community and fungi which were responsive to pesticide applications. Overall the phyllosphere was dominated by ascomycetes while a few basidiomycetous yeasts were also present and were represented by bands appearing mostly in the upper part of the gel belonging to the orders Sporidiobolales (bands 17 and 18), Cystofilobasidiales (band 19), and Filobasidiales (bands 13 and 1) (Table 1). Generally the lower part of the DGGE patterns in all treatments, which constitutes the high GC content region, was dominated by ascomycetes of the order Erysiphales (bands 2, 3, 4, 5, 10, 11, and 12). Band 15 was the most dominant band in all treatments and sequencing analysis of the clones showed highest homology (99.6%) to a *Cladosporium allii *strain (Table 1). 

A few members of the fungal community were responsive to pesticide applications. Therefore, bands 1 and 13 were present only in the samples treated with imidacloprid, especially foliage-treated samples (Figure 1). Clones associated with those bands showed highest sequence homology to a *Cryptococcus adeliensis* (97.1%) and an uncultured *Cryptococcus* clone (100%), respectively. On the contrary, band 3 disappeared from the samples which received a foliage application of imidacloprid. The single clone associated with this band showed highest sequence homology to an uncultured unclassified fungal clone (99.4%). 

Regarding metalaxyl-treated samples, band 21 was only present in the samples treated with the fungicide regardless of the application mode. Clones associated with this band showed highest sequence homology to an uncultured Saccharomyceta clone (99.6%). In addition, band 9 was stimulated in the metalaxyl-treated samples. Clones associated with this band showed highest sequence homology to a *Periconia macrospinosa *strain (99.8%).

### 3.2. Effects of Pesticides on the Bacterial Community

**Figure 3:**
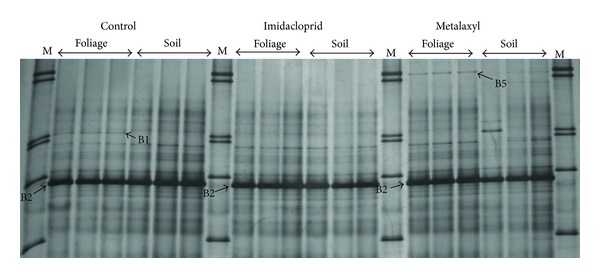
DGGE analysis of the bacterial community in the phyllosphere of pepper plants subjected to foliage or soil applications of imidachloprid, metalaxyl, or water (untreated control). Lanes designated with M correspond to a marker which contained 20 ng *μ*L^−1^ of the 16S rRNA-PCR products of each of the following bacteria appearing on the gel from top to bottom:* Pseudomonas aeruginosa, Pseudomonas *sp.,* P. putida, Flavobacterium *sp.,* Stenotrophomonas maltophilia, Xanthomonas *sp., and* Agrobacterium *sp.

**Figure 4:**
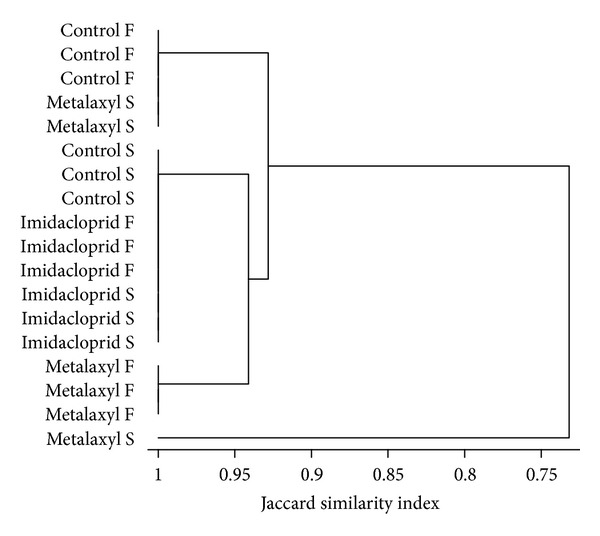
Cluster analysis (group average, Jaccard similarity index) of the banding patterns generated by DGGE fingerprinting analysis of the bacterial community.

DGGE analysis of the bacterial community showed a less complex banding pattern, compared to the fungal community fingerprints, with band numbers not exceeding 15 in any of the treatments (Figure 3). Cluster analysis separated samples into two main clusters. The first cluster contained all samples except one of the replicates of the metalaxyl soil application (Figure 4). Within the first cluster samples shared more than 90% similarity and were further separated into two sub-clusters: the first one comprising all soil controls and the two replicates of the metalaxyl soil application, while the second subcluster contained all other control, imidacloprid- and metalaxyl-treated samples. 

Clone libraries developed aimed to identify the main members of the bacterial community and bacteria responsive to pesticide applications. Fingerprints of all samples were dominated by band 2. Thus, 15 clones showing identical electrophoretic mobility with this band were sequenced. Results showed that this band was of plant origin since all clones showed highest sequence homology to chloroplast sequence of *Capsicum annum* and were excluded from further analysis. Band B5 was unique in the samples which received a foliage application of metalaxyl and the clone sequenced showed the highest sequence homology to an Enterobacteriaceae clone (100%). Similarly, band 1 was present only in the control samples and the clone associated with this band showed the highest sequence homology with a *Propionibacterium* sp. strain (100%).

## 4. Discussion

Microorganisms inhabiting the aerial parts of crops are commonly exposed to pesticides either directly through foliage applications or indirectly through soil application. The latter is valid only for systemic pesticides. Recent studies provided clear evidence for the existence of a previously unknown microbial diversity on the plant phyllosphere and the involvement of epiphytic microbes on a wide array of important services [1, 27]. Little is known regarding the interactions of pesticides and epiphytic microorganisms. We investigated the impact of two systematic pesticides and their mode of application (soil versus foliage) on the community structure of fungi and bacteria on the phyllosphere of pepper plants. 

Overall, DGGE analysis revealed a well-established fungal community and a less complex bacterial community. On the one hand, this is against the general perception that bacteria constitute the main microbial group on plant phyllosphere [8]. On the other hand, this finding is not surprising considering that pepper plants were grown under greenhouse conditions which favour the rapid establishment of fungal pathogens on aerial plant parts. In line with this are the results of the clone libraries which verified that the fungal community on the phyllosphere of pepper plants was dominated by ascomycetes with the majority of them being putative plant pathogens. Recent reports by Jumpponen and Jones [27] also found a massive dominance of ascomycetes over other fungal phyla on the phyllosphere of temperate *Quercus macrocarpa*. Several fungal ribotypes belonged to the family of Erysiphaceae whose members are the causal agents of powdery mildew in different crops like sugar beet, beetroot (*Erysiphe betae*) [28], and brassicas (*Erysiphe cruciferarum*) [29]. Among Erysiphaceae the most common ribotype was identified as *Golovinomyces cichoracearum* which is the causal agent of powdery mildew in pumpkin, cucumber, and melon crops, while it has been found as an epiphytic fungus in several other plants [30]. The dominant ribotype in the phyllosphere of pepper plants was identified as *Cladosporium allii*, which is the causal agent of leaf blotch in leek [31]. *Cladosporium* species have been identified as very common inhabitants of plant phyllosphere [32]. All the above putative plant pathogenic fungi were not affected by the application of the two pesticides. 

Generally, pesticides induced rather subtle changes in the structure of the fungal community, with foliage application of imidacloprid inducing the most prominent changes. Little is known regarding the impact of insecticides on the microbial phyllosphere. A series of studies by Zhang et al. [15–17] showed limited effects of cypermethrin and abamectin on the fungal biomass, whereas potential effects on the structure of the fungal community were not provided. The limited impact of metalaxyl on the fungal community could be attributed to its selectivity against oomycetes [33], which were absent in the phyllosphere of pepper plants in our study. Previous *in vitro* and leaf assays showed that broad-spectrum fungicides like metiram and captan had a detrimental effect on the growth of epiphytic bacteria, fungi, and yeasts with the latter being the most sensitive group of microbes [34]. 

Foliage application of imidachloprid stimulated a *Cryptococcus adeliensis* and an uncultured *Cryptococcus* ribotype. *Cryptococcus* yeasts are common epiphytes [32, 35] and members of this genus are known to effectively transform phenolics [36] and benzene [37]. Thus it is probable that the stimulation of *Cryptococcus *yeasts on the phyllosphere of pepper plants could be attributed to their involvement in the degradation of imidacloprid. Further studies will aim to isolate such strains and evaluate their degrading capacity against imidacloprid. Regarding metalaxyl, its application, either through soil or foliage, stimulated a *Periconia macrospinosa* and an uncultured ribotype belonging to the unranked taxon of Saccharomyceta. *Periconia macrospinosa *has been identified as a common plant endophyte in several studies [38, 39]. 

Overall, pesticide application induced only minor changes in the structure of the bacterial community with the exception of one of the metalaxyl soil-treated samples which diverged from all the other samples. Previous studies by Gu et al. [18] showed that the application of the fungicide enostroburin induced substantial changes in the bacterial community. A few members of the bacterial community were responsive to pesticide application. Thus foliage application of metalaxyl stimulated the appearance of an Enterobacteriaceae ribotype. This family includes several pathogens found in fresh salads destined for human consumption such as *Salmonella, Escherichia, Yersinia* [6]; plant pathogens like *Erwinia* [40]; and human pathogens like *Klebsiella *[41]. The stimulation observed could be related to the involvement of this Enterobacteriaceae ribotype in the degradation of metalaxyl; however further studies are required to confirm this. In a similar study Gu et al. [18] suggested a putative role of another Enterobacteriaceae ribotype (*Pantoea* sp.) in the degradation of the fungicide enostroburin in the phyllosphere of wheat. Members of Enterobacteriaceae belonging to the genus *Enterobacter* or *Klebsiella* have shown enhanced degrading capacities against the organophosphate chlorpyrifos [42, 43]. In contrast, pesticide application appeared to suppress a *Propionibacterium* ribotype which was present in the untreated plants. Bacteria of this genus were previously identified as members of the phyllosphere community in *Magnolia grandiflora* plants [44] and as endophytes in potatoes [45].

Generally, the DGGE screening of clone libraries followed in our study revealed the detection of two or more different ribotypes represented by the same DGGE band in one-third of the bands analyzed via cloning. This has been identified as an inherent problem of the DGGE fingerprinting method and it is commonly observed when complex microbial communities are studied [46, 47]. Further analysis of the clones obtained by the bacterial community showed that the major band of the fingerprint was related to the chloroplasts of pepper plants. Irrespective of the method employed to isolate DNA from epiphytic microbial communities, the extracted microbial DNA is generally contaminated with chloroplasts [48, 49]. Since the chloroplast 16S shares high sequence similarity with bacterial 16S rRNA sequences [50], contamination with plant DNA poses a serious challenge for the application of PCR-based methods to profile and quantify bacterial populations in plant environments. In a similar study Hunter et al. [51], using the same primer pair as in our study, also detected several sequences of chloroplast origin in their clone libraries of the phyllosphere bacterial community in lettuce.

## 5. Conclusions

Our study provides first evidence that the application of pesticides, either directly on foliage or through soil, induced rather mild changes on the structure of the fungal and bacterial communities. Further studies will aim to isolate and characterize fungal strains which were stimulated upon pesticide application and identify their true ecological role and interactions with the pesticides studied.

## Figures and Tables

**Table 1:** Identity of selected DGGE bands from clones obtained for the fungal community on the pepper phyllosphere. The numbers following % sequence similarity provide the numbers of clones showing highest similarity to this specific fungal ribotype.

Band no.	Clones sequenced	Closest match from GenBank (% sequence similarity by BLAST)	GenBank Acc. no.
1	1	*Cryptococcus adeliensis* strain ZIM600 (97.1%)	FN400760
2	6	*Golovinomyces cichoracearum* isolate (99.6%)—5	AB077656
		*Podosphaera fusca* isolate (100%)—1	AB525915
3	1	Uncultured unclassified fungal clone (99.4%)	AY843157
4	4	*Golovinomyces cichoracearum* isolate UMSG1 (99.8%)—2	HM449077
		Uncultured Sordariales clone 9A6S46N (94%)—2	HQ389517
5	7	*Erysiphe cichoracearum* (99.6%)—2	AF031282
		*Lewia infectoria* (99.8%)—5	AY154692
6	2	*Alternaria alternata* voucher TC0811057 (100%)	HM013816
7	1	*Stemphylium sp.* FA-8J (100%)	JX164072
8	3	*Pyrenophora avenae* isolate 94-1b (99.8%)—2	EF452453
		*Podospora communis* strain NZ206 (99.5%)—1	EU621831
9	2	*Periconia macrospinosa* strain KS00113 (99.8%)	FJ536208
10	4	*Neoerysiphe galeopsidis *(99.8%)—3	AB498946
		Uncultured fungus clone (99.8%)—1	JF289165
11	2	*Erysiphe betae* isolate EB1-1 (99.4%)	DQ164432
12	4	*Erysiphe cruciferarum* (99.7%)	EU140958
13	2	Uncultured *Cryptococcus* clone (100%)	JF432980
14	3	*Cladosporium cladosporioides* strain M61 (99.6%)	JQ936096
15	9	*Cladosporium allii *strain CBS 101.81 (99.6%)	JN906977
16	2	Unclassified Pleosporales isolate (100%)	FN548155
17	4	*Sporidiobolus* sp. isolate FA-8H (99.8%)	JX164071
18	1	*Sporobolomyces roseus *strain IWBT-Y851 (99.8%)	JQ993392
19	2	*Itersonilia perplexans* strain JCM 10245 (99.8%)—1	AB072233
		Uncultured fungus clone (99.8%)—1	AB520396
20	2	*Dioszegia hungarica* strain JCM 9046 (99.8%)	AB049614
21	2	Uncultured Saccharomyceta clone (99.6%)	HQ211757
